# Fertility and self-rated health of migrant women of childbearing age—an analysis of moderating effects based on socioeconomic status

**DOI:** 10.1186/s12905-024-03043-w

**Published:** 2024-04-13

**Authors:** Xue Yang, Lei Xie

**Affiliations:** 1 Northeast Asian Research Center, Jilin University, Qianjin Street No. 2699, Changchun, 130012 Jilin Province China; 2 Northeast Asian Studies College, Jilin University, Qianjin Street No. 2699, Changchun, 130012 Jilin Province China

**Keywords:** Fertility, Self-rated health, Women of childbearing age, Migrant women

## Abstract

**Background:**

As fertility rates continue to decline and negative population growth emerges, China has sequentially introduced encouraging fertility policies to raise fertility levels. The impact of fertility on women’s health remains inconclusive. It is essential to explore further the correlation between fertility and the health status of 113 million migrant women of childbearing age in China.

**Objective:**

To investigate how fertility affects the health status of migrant women of childbearing age and determine if migrant women’s socioeconomic status plays a moderating role in this process.

**Methods:**

Using a nationally representative dataset from the 2018 China Migrants Dynamic Survey (CMDS), we examined the effects of fertility on the self-rated health of migrant women of childbearing age. An ordinary least squares regression model with moderating effects was used for the empirical study, and robustness tests were conducted based on the ordered probit model and propensity score matching to address endogeneity.

**Results:**

The empirical results indicated that a rise in the number of children born significantly reduces the self-rated health of migrant women of childbearing age. An increase in years of schooling and household income can significantly mitigate the negative impact of childbearing on the health of migrant women. The robustness of the above results was validated through alternative models and propensity score matching (PSM) methods. The heterogeneity analysis revealed that fertility exerts a negative impact on the health status of migrant women with rural household registration and on the health status of inter-provincial and inter-city migrant women. Further investigation found that the occurrence of childbirth during migration and an increase in the number of girls significantly negatively impacted the health status of migrant women. In contrast, the increase in the number of boys did not show a significant effect. Improving the health of migrant women of childbearing age significantly positively impacted their future childbearing intentions.

**Conclusions:**

Migrant women of childbearing age bear the dual burden of migration and childbirth. Our findings showed the rise in the number of children born and the occurrence of childbirth during migration posed greater challenges to the health status of female migrants, particularly among those with lower socioeconomic status. Government and community efforts for enhancing health among migrant women of childbearing age are recommended.

## Introduction

Data from the most recent three National Censuses indicate that China’s total fertility rate has consistently remained at or below 1.3 (1.22, 1.18, and 1.30 for the fifth to seventh National Censuses, respectively). A prolonged period of ultra-low fertility levels will challenge the long-term balanced development of population. Therefore, since 2013, the country has gradually loosened its restrictions on fertility policies. In May 2021, China implemented a three-child policy along with supportive measures, signifying a shift in China’s family planning policy from a restrictive to an inclusive approach [1].

An increase in the number of children born can have various impacts on individuals and families, an important one being the health status of the parents, particularly the women of childbearing age [2]. Most academic studies indicate that increasing the number of children can negatively impact women’s health [3, 4]. However, some studies suggest that the relationship is “U-shaped” or “J-shaped” [5–7], and some even find a positive effect [8]. The impact of fertility on health remains inconsistent in academic research, and most of the findings are based on data from developed countries. As a country in transition with rapid socioeconomic changes, China requires more detailed and in-depth research on related issues.

With the gradual advancement of urbanization in China, the number of migrants is on the rise. Based on data from the 7th National Census, the total number of migrants in China reached 376 million in 2020, representing more than one-quarter of the country’s total population. Among the migrants, the number of women of childbearing age has reached 113 million, which accounts for 30.19% of the total migrant population. According to the definition of National Health Commission of the People’s Republic of China, migrants are those who have lived in an inflow area for more than one month, whose household registration is not registered in the local district, and are over 15 years old [9].

The household registration system is vital to the Chinese people because it is closely related to their access to welfare benefits. As migrants do not have local household registration, compared to the local residents, they face significant inequities and discrimination in terms of access to social welfare and public services [10, 11]. It is difficult for the migrants to access the opportunities, benefits, social security and services in the labor market and social life of the inflow areas that the local registered population has, whose primary requirements for social services and security still a considerable extent rely on the rural areas where they originate from, so the migration experience has produced a dissipative effect on the health of migrants [12]. Interruption theory posits that the separation of couples or challenges encountered during migration can result in disruptions to fertility [13]. Migration often delays migrant women’s childbirth goals, resulting in women becoming pregnant at an older age which can have negative physical and mental health effects such as gestational diabetes, hypertension, and perinatal depression [14, 15]. Due to the challenges faced by migrant women of childbearing age in accessing adequate welfare benefits during migration, coupled with the heightened risk of advanced maternal age childbirth. It is of great practical significance to study the impact of fertility on the health of migrant women of childbearing age.

Socioeconomic factors play a crucial role in influencing the health of migrants and also have an effect on women’s fertility. Education and income serve as fundamental indicators of an individual’s socioeconomic status [16]. According to the resource amplification theory, the health benefits of education follow a “Matthew effect,” whereby the strong become stronger, and the weak become weaker [17]. Absolute income hypothesis also suggests that population health improves with average income [18], empirical studies have consistently shown the positive effect of income on an individual’s health [19]. As women’s education levels increase, their concept of childbearing changes, which in turn affects their fertility [20]. According to Becker’s Quantity-quality trade-off theory, the higher education status of women can be expected to be related to a higher demand for child quality (educational attainment of the child); this would have led families to substitute quality for quantity by having fewer children and investing more in each child [21]. Similarly, as the income of women of childbearing age increase, increasing opportunity costs of childbearing contribute to a reduced demand for children. As the value of time rises, if women have more children, they will lose money. When mothers prefer labor force participation for more income rather than spend more time caring for their children, fertility levels decrease [22]. Socioeconomic status may play a moderating role in the relationship between fertility and women’s health.

Based on the above context, this study utilizes data from the 2018 China Migrants Dynamic Survey (CMDS) conducted by the National Health Commission of the People’s Republic of China to examine the relationship between fertility and the health status of migrant women of childbearing age and if there is a moderating effect of migrant women’s socioeconomic status in this process.

## Materials and methods

### Data source

This paper utilizes data from the 2018 China Migrants Dynamic Survey (CMDS) of the National Health Commission of the People’s Republic of China. This survey data is an annual large-scale nationwide sample survey of the migrants conducted by the National Health Commission since 2009, covering 31 provinces, cities and autonomous regions, and the Xinjiang Production and Construction Corps in the inflow areas where the migrants is concentrated, employing a stratified, multi-stage, probability proportional to size (PPS) sampling method. Since the study subject of this paper is women of childbearing age between 15 and 49 years old, the original sample size obtained was 65,725, the final sample size used for the study was obtained as 51,286 by eliminating the samples that were missing values.

### Variables

The dependent variable in this paper is the self-rated health of migrant women of childbearing age; we constructed this variable by analyzing the responses to the “How is your health status?” question from the CMDS survey questionnaire. The answers include healthy, basically healthy, unhealthy, but able to take care of oneself, and not able to take care of oneself, with the options being allocated in the sequence 4 = healthy, 3 = basically healthy, 2 = unhealthy, but able to take care of oneself, and 1 = not able to take care of oneself.

The key explanatory variable in this paper is fertility, which is measured by the total number of births. In the CMDS, it was obtained by the question, “How many children have you had?” A response of 0 indicates no children; the maximum number of children reported was 7, representing a continuous variable.

The moderating variables in this study are the socioeconomic status of the participants. Years of education and household income were used as proxy variables to measure socioeconomic status, years of education were converted based on the level of education attained^1^, and the household income was logarithmically transformed.

Control variables include three levels of dimensions: personal characteristics, health characteristics, and migration characteristics. Personal characteristics variables include age, ethnicity, household registration, and marital status; health characteristics variables comprise whether an individual has medical insurance, whether an individual has received reproductive health education, whether an individual has established a resident health record, and whether an individual has contracted with a local doctor; migration characteristics variables encompass the distance of migration, the reason for migration, and the area of migration.

### Empirical methods

This manuscript employs moderated ordinary least squares (OLS) regression analysis by reference to Wen et al. as follows [23]:

1 $$Y = \beta_{0} + \beta_{1}{X}_{1} + \beta_{2} M + \beta_{3}M \times X_{1} + \beta_{4} \; controls + \epsilon$$

In Eq. (1), Y denotes self-rated health, β_0_ is a constant term, β_i_ is the coefficients of the variables, X_1_ is fertility, M represents a socioeconomic status variable, controls represent control variables, and ε is the random interference term.

### Empirical results

#### Descriptive statistical analysis

Table 1 indicates that the overall health status of migrant women of childbearing years is relatively favorable. More than 97% of these women perceive themselves to be in a healthy or basically healthy state, with a relatively small proportion of women reporting as unhealthy or cannot take care of themselves. As the number of children increases, the proportion of unhealthy women of childbearing age gradually rises, while the proportion of healthy or basically healthy women of childbearing age gradually declines. Moreover, the self-rated health of women of childbearing age is found to be negatively associated with the number of children born preliminary.

**Table 1:** Number of children born and health status (unit: %)

Number of children born	Not able to take care of oneself	Unhealthy, but able to take care of oneself	Basically healthy	Healthy
0	0.052	0.734	7.051	92.163
1	0.028	1.058	9.606	89.308
2	0.025	1.615	10.768	87.592
3 and above	0.076	3.170	13.713	83.040

*Data source* 2018 CMDS data.

Table 2 presents the means or frequencies of the main variables, with descriptive statistical analyses grouped based on whether childbearing occurred during this migration. The results indicate that the health status of migrant women of childbearing age is relatively good, with a mean value of 3.871. Furthermore, the average number of births is 1.428, significantly lower than the replacement level of 2.1. Regarding personal characteristics, the average number of years of education is 10.201 years; The proportion of women aged 25–34 is 47.613%, accounting for the largest share, followed by 35–44 years old, 45–49 years old, and 15–24 years old, respectively; 91.0% of women are Han Chinese, 82.9% are in agricultural household registration, and 96.1% are in marital status.

Among the health characteristics, it was found that 93.7% of women have at least one kind of medical insurance. Additionally, 52.7% of women have received reproductive health education within their communities or work units. Furthermore, 30.9% of women have established resident health records, and only 13.6% of women have contracted with a local doctor.

In terms of migrant characteristics, the largest share of the migration distance is inter-provincial migration, which is 48.602%, followed by inter-city within provinces and inter- county within cities. Concerning reasons for migration, 77.676% of women of childbearing age migrated for work and business, accounting for the largest proportion, followed by migration for marriage and other reasons. As for the areas of migration, 45.123% of women of childbearing age migrated to the eastern region of China, whereas 29.193% have migrated to the western region and 19.690% to the central region, and the least 5.994% to the northeast region.

The sample was classified based on whether or not childbearing occurred during the migration. The results indicate that women of childbearing age who gave birth during the migration have higher mean values for health status, the number of children born, years of education, and household income compared to those who did not give birth during this migration; moreover, the percentage of women who were married, received health education, established health records, and contracted with local doctors are also higher; in contrast, the percentage of Han Chinese and agricultural households are lower.

Compared to the women who did not conduct fertility behavior during migration, the proportion of women who conducted fertility behavior during migration is larger in the younger age group (15–44 years old) and lower in the senior age group (45–49 years old); the proportion of inter-city within the province is higher, while the inter-provincial migration and inter-county within the cities are lower among the migration distance; the percentage of individuals migrating for marriage and other reasons is more significant, while the proportion of that migrating for work and business is relatively more minor; the proportion of those who migrate to the eastern and western regions is relatively high, and those who migrate to the central and northeastern regions are relatively low.

**Table 2:** Descriptive statistical analysis

Variables	Definition	Total	Birth migration	No birth migration
		*N* = 51,286	*N* = 23,238	*N* = 28,048
Dependent variable				
Self-rated health	1 = Not able to take care of oneself; 2 = Unhealthy, but able to take care of oneself; 3 = Basically healthy; 4 = Healthy	3.871	3.889	3.856
Independent variable				
Fertility	Number of children born	1.428	1.556	1.322
Moderating Variables				
Education level	Years of education	10.201	10.949	9.582
Household income	Logarithmic processing	8.823	8.877	8.777
Personal characteristics				
Age	15–24 years	7.427%	9.700%	5.544%
	25–34 years	47.613%	59.433%	37.821%
	35–44 years	32.313%	26.629%	37.022%
	45–49 years	12.647%	4.239%	19.613%
Ethnicity	1 = Han Chinese; 0 = Minority	0.910	0.904	0.915
Hukou	1 = Agricultural; 0 = Non-agricultural	0.829	0.808	0.847
Marital Status	1 = In marriage; 0 = Not in marriage	0.961	0.979	0.946
Health characteristics				
Medical insurance	1 = With insurance; 0 = Without insurance	0.937	0.935	0.940
Health education	1 = Received; 0 = Not received	0.527	0.564	0.497
Health record	1 = Established; 0 = Not	0.309	0.334	0.288
Local doctor	1 = Contracted; 0 = Not contracted	0.136	0.155	0.120
Migrant characteristics				
Migration distance	Inter-provincial	48.602%	47.947%	49.144%
	Inter-city	33.543%	34.547%	32.712%
	Inter-county	17.855%	17.506	18.144%
Reasons for migration	Work and business	77.676%	70.247%	83.831%
	Marriage	20.241%	27.545%	14.190%
	Other reasons	2.082%	2.208%	1.979%
Migration areas	Eastern region	45.123%	46.803%	43.732%
	Central region	19.690%	18.810%	20.419%
	Western region	29.193%	29.301%	29.104%
	Northeast region	5.994%	5.086%	6.746%

### Baseline regression analysis

Table 3 shows the results of the baseline regression analyses. The first model incorporated the key independent variable. The second model incorporated the moderating variable of years of education, the third model incorporated the moderating variable of household income, and the fourth model included both moderating variables. Models 1 to 4 are all at the 1% significance level, the results reveal that the increased number of children born significantly decreases the health status of migrant women of childbearing age.

Models 2 to 4 indicate that the interaction term between the socioeconomic status variables (years of education and household income) and the number of children born positively affects the self-rated health of migrant women of childbearing age; this suggests that the socioeconomic status of migrant women positively moderates their health status and that as their socioeconomic status increases, the negative impact of having more children on their health is significantly reduced.

Regarding control variables, among the personal characteristics, it is found that the health status of women in the other age groups is poorer compared to women in the 15–24 age group; Han Chinese, agricultural households, and married women show better health statuses. Among the health characteristics variables, it is observed that possessing medical insurance, receiving reproductive health education, establishing health records, and contracting with a local doctor can significantly improve the health status of migrant women of childbearing age. Among the variables of migration characteristics, compared to inter-provincial women, the health status of inter-city and inter-county women is poorer; women who migrate for marriage and other reasons are less healthy than those who migrate for work and business. Compared to women who migrate to eastern China, women who migrate to the western and northeastern regions have poorer health status, and women who migrate to the central region have better health status.

**Table 3:** Baseline regression results

Variables	Model 1		Model 2		Model 3		Model 4	
	*N* = 51,286		*N* = 51,286		*N* = 51,286		*N* = 51,286	
	Estimate	Std.error	Estimate	Std.error	Estimate	Std.error	Estimate	Std.error
Fertility	-0.010***	0.003	-0.034***	0.009	-0.133***	0.042	-0.120***	0.041
Years of education			0.004***	0.001			0.003**	0.001
Fertility×education			0.003***	0.001			0.003***	0.001
Household income					0.024***	0.007	0.024***	0.007
Fertility×income					0.014***	0.005	0.010**	0.005
Age group								
15–24 years								
25–34 years	-0.023***	0.005	-0.029***	0.005	-0.025***	0.005	-0.029***	0.005
35–44 years	-0.099***	0.006	-0.091***	0.006	-0.095***	0.006	-0.090***	0.006
45–49 years	-0.217***	0.008	-0.194***	0.008	-0.208***	0.008	-0.191***	0.008
Ethnicity	0.043***	0.007	0.032***	0.007	0.037***	0.007	0.029***	0.007
Hukou	-0.003	0.004	0.018***	0.005	0.005	0.004	0.021***	0.005
Marital status	0.034***	0.010	0.030***	0.010	0.017*	0.010	0.016	0.010
Medical insurance	0.018**	0.007	0.013*	0.007	0.013*	0.007	0.010	0.007
Health education	0.031***	0.003	0.027***	0.003	0.030***	0.003	0.027***	0.003
Health record	0.021***	0.004	0.019***	0.004	0.021***	0.004	0.019***	0.004
Local doctor	0.019***	0.005	0.019***	0.005	0.020***	0.005	0.020***	0.005
Migrant distance								
inter-provincial								
inter-city	-0.004	0.004	-0.008*	0.004	-0.004	0.004	-0.007	0.004
Inter-county	-0.012**	0.005	-0.015***	0.005	-0.007	0.005	-0.010*	0.005
Migration reasons								
Work and business								
Marriage	-0.047***	0.005	-0.047***	0.005	-0.042***	0.005	-0.043***	0.005
Other reasons	-0.073	0.015	-0.074***	0.015	-0.069***	0.015	-0.071***	0.015
Migration areas								
Eastern region								
Central region	0.003	0.011	0.006	0.011	0.019*	0.011	0.020*	0.011
Western region	-0.070***	0.016	-0.052***	0.016	-0.045***	0.016	-0.034**	0.016
Northeast region	-0.121***	0.018	-0.106***	0.018	-0.092***	0.018	-0.084***	0.018
Provincial control	Yes		Yes		Yes		Yes	
Constant term	3.896***	0.015	3.839***	0.021	3.677***	0.068	3.639***	0.06
R2_a	0.050		0.055		0.056		0.059	

*Note* *P < 0.10; **P < 0.05; ***P < 0.01.

#### Robustness test

##### Replacement model

As the dependent variable is an ordinal variable, the regression model is replaced with an ordered probit (Oprobit) model for robustness testing. The model 5 incorporated the key explanatory variable. Models 6 and 7 contain the moderating variables of years of schooling and household income, respectively. Model 8 includes the two moderating variables. The findings in Table 4 demonstrate that the key explanatory variable remains significantly negative, similar to the results in Table 3; this implies that as the number of children born increases, the health status of migrant women of childbearing age significantly declines.

In addition, the interaction term between the years of education and fertility had a significant positive impact on self-rated health in both Model 6 and Model 8, indicating that the impact of fertility on health status is positively moderated by years of education. Similarly, the interaction term between household income and fertility in Model 7 has a significantly positive moderating effect on health status. However, the household income moderating effect was not significant in Model 8. In general, the robustness of the conclusions can be obtained through the method of replacement model.

**Table 4:** Robustness tests (Oprobit model)

Variables	Model 5		Model 6		Model 7		Model 8	
	*N* = 51,286		*N* = 51,286		*N* = 51,286		*N* = 51,286	
	Estimate	Std.error	Estimate	Std.error	Estimate	Std.error	Estimate	Std.error
Fertility	-0.039***	0.011	-0.052*	0.027	-0.255**	0.125	-0.241*	0.123
Years of education			0.026***	0.005			0.021***	0.005
Fertility×education			0.006**	0.003			0.005**	0.003
Household income					0.108***	0.024	0.094***	0.024
Fertility×income					0.025*	0.014	0.022	0.014
Control variables	Yes		Yes		Yes		Yes	
Provincial control	Yes		Yes		Yes		Yes	

*Note* *P < 0.10; **P < 0.05; ***P < 0.01.

### Propensity score matching

As fertility is a decision made by women of childbearing age, it is a non-random event, and various other factors can influence fertility. Therefore, the estimated coefficients may be biased due to the self-selection problem. To further ensure the robustness of the results, this study adopts the propensity score matching method (PSM) to deal with the endogeneity issue, which can effectively address the problems of omitted variables and sample self-selection bias. The fundamental concept of PSM is to construct a counterfactual framework and an approximate “randomized experiment” to eliminate significant bias resulting from the observable characteristics of the treatment and control groups and calculate the average treatment effect.

Given that the independent variable in this paper is continuous, we have categorized the samples whose number of children born exceeds the mean value of 1.428 as the treatment group, and those below 1.428 as the control group. We further examined the robustness of the results through four matching methods: K-nearest neighbor matching, radius matching, kernel matching, and intra-caliper K-nearest neighbor matching.

Table 5 presents the average treatment effects of fertility on the health of migrant women of childbearing age under different matching methods. We used the Bootstrap self-sampling methods to adjust the possible bias because of single matching. The results in Table 5 indicate that the average treatment effects obtained from various matching methods are significant at the 1% level. Migrant women with an actual number of children born above the mean value have a significantly worse self-rated health status of 0.014–0.017 compared to those born below the mean value. The ATT values are consistent across different matching methods, further supporting the robustness of the results. This robustness test does not incorporate moderating variables and interaction terms.

**Table 5:** Robustness tests (Propensity score matching)

Matching method	Average treatment effect	Bootstrap std.error	T-value
K-Nearest neighbor matching	-0.017	0.005	-3.51***
Radius matching	-0.014	0.004	-3.39***
Kernel matching	-0.014	0.004	-3.43***
Intra-caliper k-nearest neighbor matching	-0.017	0.004	-4.16***

*Note* K nearest neighbor matching set K = 4; radius matching set caliper value = 0.01; kernel function and bandwidth of kernel matching use their default values; intra-caliper K-nearest neighbor matching set K = 4, caliper value = 0.01; Bootstrap sampling number is 100.

### Heterogeneity analysis

The analysis above reveals that an increase in the number of children born significantly reduces the self-rated health of migrant women of childbearing age. However, there are notable distinctions among the childbearing of different groups of women of childbearing age, leading to varying impacts on women’s health. Tables 6 and 7 explore the heterogeneity in the impacts of fertility on the health of migrant women of childbearing age with varying household registration and migration distance, respectively.

As a result of China’s dualistic economic institution, there exists a notable difference in the social resources and welfare accessible to women of childbearing age during the process of childbirth. Table 6 shows that an increase in the number of children significantly reduces the self-rated health of rural migrant women. However, this effect is insignificant for migrant women with non-agricultural household registration.

**Table 6:** Fertility and health of migrant women of childbearing age in different household registration

Variables	Rural *N* = 42,533		Urban *N* = 8753	
	Estimate	Std.error	Estimate	Std.error
Fertility	-0.127***	0.046	-0.093	0.095
Moderating variables	Yes		Yes	
Control variables	Yes		Yes	
Provincial control	Yes		Yes	
Constant term	3.676***	0.078	3.576***	0.128
R^2^_a	0.060		0.056	

*Note* *P < 0.10, **P < 0.05, ***P < 0.01.

This paper further conducts group regression based on the migrant distance; the results show that further of the migration distance, the negative impact of fertility on women’s health is more serious. Specifically, an increase in the number of children born can significantly reduce the health status of women who migrate inter-provincially and inter-city. In contrast, there is no significant adverse effect of fertility on the health of inter-county migrant women. This situation could be attributed to the notion that as a woman migrates farther away from her place of origin, she may receive less support from her family and encounter more incredible difficulty in obtaining reproductive-related benefits.

**Table 7:** Fertility and health of women of childbearing age with different migrant distance

Variables	Inter-provincial *N* = 24,926		Inter-city *N* = 17,203		Inter-County *N* = 9157	
	Estimate	Std.error	Estimate	Std.error	Estimate	Std.error
Fertility	-0.129**	0.053	-0.161**	0.082	0.032	0.096
Moderating variables	Yes		Yes		Yes	
Control variables	Yes		Yes		Yes	
Provincial control	Yes		Yes		Yes	
Constant term	3.737***	0.083	3.634***	0.132	3.306***	0.176
R2_a	0.046		0.070		0.076	

*Note* *P < 0.10, **P < 0.05, ***P < 0.01.

### Further analysis

The concept of fertility includes more than one indicator of the number of children born. Next, we investigated the impacts of different fertility behaviors on the health of migrant women of childbearing age in four aspects: whether to give birth during this migration, the number of boys born, the number of girls born, and the gender structure of fertility (number of boys divided by the number of girls).

The findings presented in Table 8 demonstrate that giving birth during migration significantly weakens the self-rated health of women of childbearing age, indicating that migrant women would face additional challenges if they chose to give birth during migration because it is difficult for them to access the same welfare benefits that local women can more easily obtain. Ultimately, this fertility behavior hurts the health of migrant women of childbearing age.

Furthermore, models 10 to 12 reveal that a rise in the number of female children considerably diminishes the self-rated health of mothers. Conversely, the increase in the number of male children and the proportion of boys in the gender structure does not negatively impact the health of migrant women of childbearing age; meanwhile, the corresponding coefficient values are positive. The potential reason for this phenomenon may be the persistence of the “son preference” among the migrants. With the birth of more boys in migrant families, mothers can receive better healthcare and nutrition support from the family. Furthermore, the birth of male children can provide mothers with greater psychological satisfaction, ultimately resulting in no significant negative impact on their self-rated health.

**Table 8:** Effects of different fertility behaviors on the health of migrant women of childbearing age

Variables	Model 9 *N* = 51,286		Model 10 *N* = 47,471		Model 11 *N* = 47,471		Model 12 *N* = 27,363	
	Estimate	Std.error	Estimate	Std.error	Estimate	Std.error	Estimate	Std.error
Birth migration	-0.009**	0.003						
Number of boys			0.002	0.003				
Number of girls					-0.005**	0.003		
Fertility structure							0.005	0.005
Control variables	Yes		Yes		Yes		Yes	
Provincial control	Yes		Yes		Yes		Yes	
Constant term	3.457***	0.036	3.426***	0.038	3.430***	0.038	3.444***	0.050
R2_a	0.058		0.060		0.060		0.060	

*Note* *P < 0.10, **P < 0.05, ***P < 0.01.

Given the continued decline in China’s fertility rate in recent years, coupled with the substantial size of the migrant population of childbearing age, increasing the fertility rate of the migrants has become a crucial factor in improving the overall fertility level. The subsequent content centers on the impact of the health status of migrant women of childbearing age on their future fertility intentions. The binary dependent variable is whether or not migrant women plan to give birth in the following two years. The key independent variable is the self-rated health status of migrant women of childbearing age. Table 9 employs Logit and Probit models to perform the empirical analysis. The results demonstrate that enhancement in health status significantly raises the probability of migrant women’s intention to have children within the next two years.

As increasing the number of children born significantly negatively impact the health status of women of childbearing age, the negative health effect is one of the reasons that women forgo giving birth to a higher parity. This situation is manifestly incongruous with China’s intention to elevate the national fertility levels.

The findings presented in Table 9 can enlighten Chinese policymakers. Specifically, the government ought to fully consider women regarding the negative health effect of fertility-related actions when it attempts to increase the overall fertility level. If migrant women of childbearing age could receive adequate support and assistance from their families and society during the phases of childbirth and child-rearing, and their health status is optimally protected, then it would significantly increase their desire to have more children in the future.

**Table 9:** Self-rated health and future childbearing intentions of mobile women of childbearing age

Variables	Logit *N* = 50,002			Probit *N* = 50,002	
	Estimate		Std.error	Estimate	Std.error
Self-rated health	0.106*	0.059		0.058*	0.031
Control variables	Yes			Yes	
Provincial control	Yes			Yes	
Constant term	-4.644***	0.690		-2.806***	0.385

*Note* *P < 0.10, **P < 0.05, ***P < 0.01.

## Discussion

There has been much academic research on the impact of fertility on the health of mothers, but the conclusions are not consistent. Some studies have reported a negative impact of fertility on maternal health, while others have observed a non-linear or positive relationship between the two variables [3–8, 24–26]. Most research has focused on the health of older mothers, with limited attention given to how fertility influences the health of migrant women of childbearing age. Given the large number of migrant women of childbearing age in China and the fact that they often suffer from the inequality and discrimination caused by the household registration system, this population group deserves more attention. Based on the data derived from the 2018 China Migrants Dynamic Survey (CMDS), this paper examines the influence of fertility on the self-rated health of migrant women of childbearing age, examining how fertility affect the health of mothers through a new research target.

The finding suggests that there is a significant negative correlation between the number of children born and the self-rated health of migrant women of childbearing age; in addition, an elevation in the socioeconomic status can act as a positive moderator and mitigate the negative impact of fertility on the health of migrant women. Besides, the results of the heterogeneity analysis indicate that fertility has varying impacts on the health of different categories of migrant women. The number of children born negatively affects the health of rural hukou, inter-provincial, and inter-city migrant women. In contrast, it does not significantly affect the health of urban hukou and inter-county migrant women.

It is crucial to fully consider the negative effect of childbearing on the health status of women of childbearing age, with the health loss resulting from high parity being one of the reasons leading to women’s reluctance to give birth to more children. Regarding the socioeconomic status of migrant women, it is notable that improving the level of education and household income among migrant women can considerably mitigate the adverse impact of fertility on their health status; meanwhile, the negative effect of fertility on rural women’s health is comparatively more prominent. Thus, the health status of migrant women of childbearing age with lower socioeconomic status should receive more attention.

Further investigation into the effects of various fertility behaviors on the health of migrant women, we found that giving birth during migration and an increase in the number of girls had a significant negative impact on the self-rated health of migrant women. Conversely, an increase in the number of boys and the proportion of boys in the gender structure did not significantly affect maternal health.

The dualistic urban-rural economic structure has long hindered the migrants from enjoying the same benefits as the local household population, resulting in more challenging working and living environments and significant difficulties in accessing healthcare [27], making them a vulnerable group. The migrants are significant contributors and participants in China’s economic and social development. However, they still face the harsh reality of marginalization and numerous disparities, particularly concerning healthcare [28].

In addition to the impact of the increase in the number of children born on the health of migrant women of childbearing age, other factors can make the occurrence of childbearing during migration have an impact on women’s health. First, migrants face challenges in accessing opportunities, benefits, social security, and services in inflow areas due to the lack of local household registration [10, 11]. Second, the migration experience postpones migrant women’s fertility intentions, thereby elevating the probability of them giving birth at an advanced age [29]. The adverse health consequences associated with advanced maternal age childbirth have been extensively documented in the medical domain [14, 15]. Third, most of the migrant population are engaged in jobs that are not very good, belonging to the bottom class, such as some service industry jobs, and the longer the duration of their occupation, the poorer their health condition [11, 30]. Migrant women of reproductive age, in particular, face the dual challenges of migration and childbirth. Therefore, their health status demands greater attention, as their fertility during migration will induce more inconvenience and obstacles.

Moreover, improved self-rated health of migrant women of childbearing age significantly increased the probability of giving birth in the following two years. With the declining fertility rate in China, improving the health status of migrant women of childbearing age and compensating for their health loss during childbirth is one of the strategies to raise future fertility levels. For instance, the government could introduce specific policies and regulations that offer targeted healthcare and financial aid to migrant women of childbearing age during childbirth. Such measures would not only alleviate migrant women’s anxieties concerning potential health loss associated with childbirth but would also potentially yield benefits in terms of increasing future fertility rates.

## Conclusions

An increase in the number of children significantly reduces the self-assessed health status of migrant women of childbearing age, and an increase in socioeconomic status positively moderates this negative effect; the impact of childbearing on the health of rural and long-distance migrant women is more significant than that of urban and short-distance migrant women. In addition, the occurrence of childbirth during the period of migration significantly reduces the health of migrant women; in terms of the sex of the children born, an increase in the number of born female children significantly reduces the health of the mothers, while an increase in the number of born male children does not have a significant effect. An increase in the health status of migrant women of childbearing age significantly enhances their future desire for childbearing.

## Data Availability

The datasets analyzed during the current study are available from the 2018 China Migrants Dynamic Survey of the National Health Commission of the People’s Republic of China. Application for use through research institution.

## Footnotes

1 The conversion criteria between education level and years of education: according to the “education level” option “illiteracy,” “elementary school,” “junior high school,” “high school/secondary school,” “college,” “undergraduate,” “postgraduate,” respectively converted to 0 years, 6 years, 9 years, 12 years, 15 years, 16 years, 19 years.
